# Triggered reversible phase transformation between layered and spinel structure in manganese-based layered compounds

**DOI:** 10.1038/s41467-019-11195-9

**Published:** 2019-09-02

**Authors:** Mi Ru Jo, Yunok Kim, Junghoon Yang, Mihee Jeong, Kyeongse Song, Yong-Il Kim, Jin-Myoung Lim, Maenghyo Cho, Jae-Hyun Shim, Young-Min Kim, Won-Sub Yoon, Yong-Mook Kang

**Affiliations:** 1 Department of Energy and Materials Engineering, Dongguk University-Seoul, Seoul, 04620 Republic of Korea; 2 Department of Energy Science, Sungkyunkwan University, Suwon, 16419 Republic of Korea; 3 Korea Research Institute of Standards and Science (KRISS), Daejeon, 34113 Republic of Korea; 4 Department of Mechanical and Aerospace Engineering, Seoul National University, Seoul, 08826 Republic of Korea; 5 Department of Advanced Materials and Energy Engineering, Dongshin University, Naju, 58245 Republic of Korea; 6 Center for Integrated Nanostructure Physics, Institute for Basic Science (IBS), Suwon, 16419 Republic of Korea; 7 Present Address: Department of Materials Science and Engineering, Korea University, Seoul, 02841 Republic of Korea

**Keywords:** Batteries, Batteries, Batteries, Batteries

## Abstract

Irreversible phase transformation of layered structure into spinel structure is considered detrimental for most of the layered structure cathode materials. Here we report that this presumably irreversible phase transformation can be rendered to be reversible in sodium birnessite (Na_*x*_MnO_2_·*y*H_2_O) as a basic structural unit. This layered structure contains crystal water, which facilitates the formation of a metastable spinel-like phase and the unusual reversal back to layered structure. The mechanism of this phase reversibility was elucidated by combined soft and hard X-ray absorption spectroscopy with X-ray diffraction, corroborated by first-principle calculations and kinetics investigation. These results show that the reversibility, modulated by the crystal water content between the layered and spinel-like phases during the electrochemical reaction, could activate new cation sites, enhance ion diffusion kinetics and improve its structural stability. This work thus provides in-depth insights into the intercalating materials capable of reversible framework changes, thereby setting the precedent for alternative approaches to the development of cathode materials for next-generation rechargeable batteries.

## Abstract (web summary)

The irreversible layered-to-spinel phase transformation is detrimental for many cathode materials. Here, the authors show that reversibility can be realized in crystal water containing sodium birnessite by controlled dehydration, leading to enhanced ion diffusion kinetics and improved structural stability.

## Introduction

Layered-structured oxides have been regarded as the conventional cathode materials in rechargeable batteries due to their advantageous properties and good electrochemical performances. Generally, layered-structure oxides tend to transform into a defective spinel-like structure during alkali ion extraction, a process which seems especially severe for manganese-based layered materials. This phase transformation from layered to spinel structure is induced by the rearrangement of transition metal ions and alkali ions^1–4^. This transformation is normally considered irreversible and therefore should be prevented. Indeed, most of the research undertaken to date have focused solely on suppressing this irreversible phase transformation (e.g. by partial substitution of manganese with other transition metals), while there was little success in making this structural transformation highly reversible^5^. However, we recognize that this transformation may be exploited to activate new cation sites and modulate the redox behaviors, which may ultimately enable to utilize the full potential of layered cathode materials^6–8^. Exploration along this theme may broaden the applicability of layered structure oxides as electrode materials and develop new routes for innovating energy storage technologies.

Recently, Na-birnessite (Na-bir) has attracted plenty of interests due to its unique structure containing crystal water in the Na layer^9–12^. Normally, Na-bir is a layered-structure composed of edge-sharing MnO_6_ octahedra, where Mn^4+^ cations act as redox centers. A charge deficit in the slab arises from the presence of Mn^3+^ cations in the interlayers and/or a manganese vacancy in the octahedral center. This deficit is normally compensated by the presence of interlayer hydrolyzable cations^13–16^. Here, the crystal water in the Na layer differentiates Na-bir from other layered-structure oxides. Dehydration of Na-bir would increase the number of vacancies in the transition metal layers because of the layer-to-interlayer migration of Mn^3+^ cations, resulting in the formation of triple corner-sharing Mn. The migration of Mn^3+^ cations into the interlayer also releases the steric strain associated with the Jahn–Teller distortion of the MnO_6_ octahedra^13^. These features stimulated couple of previous studies into the crystallographical and chemical properties of Na-bir, although its pristine structure and structural dynamics during oxidation/reduction remain unclear, especially regarding how those aforementioned features affect or dominate the redox processes and the associated phase transformation. Specifically, by elucidating how the layered structure is rearranged and identifying the cation sites that can be activated during oxidation/reduction, we aim to overcome the performance limitation of layered cathode materials through the reversible transformation between the layered and spinel phases. Hence, in this paper, we investigate how the structural dynamics of Na-bir during charge/discharge are affected by reducing the content of crystal water. The results here show that partial dehydration renders the Na-bir structure flexible, thereby enabling the reversible phase transformation between the layered and spinel-like structure.

## Results

### Structural characterization

**Fig. 1:**
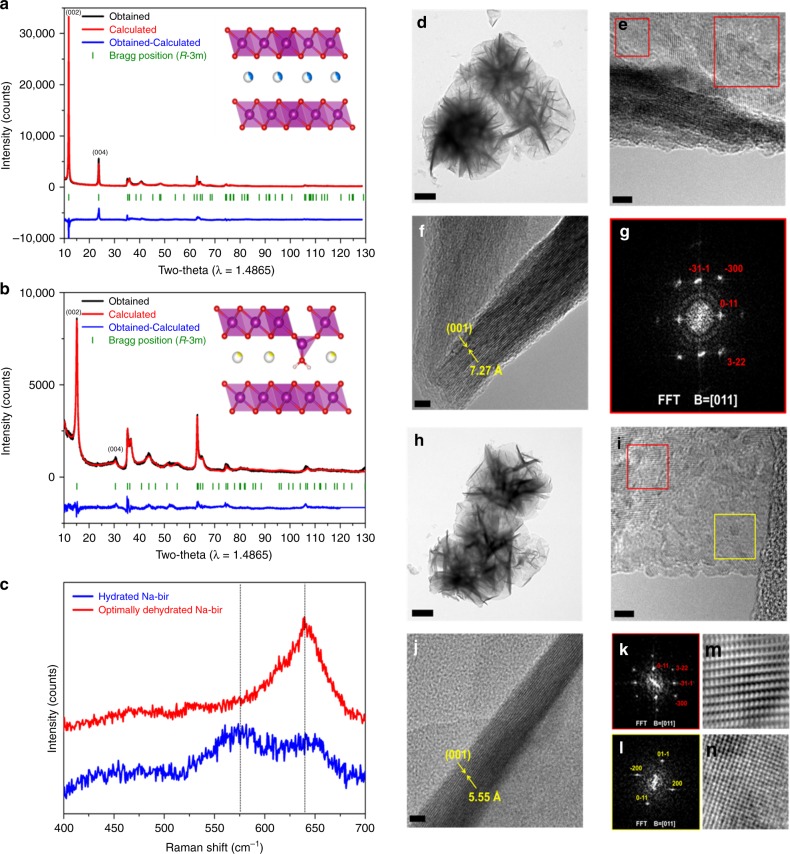
Structural characterizations of hydrated and partially dehydrated Na-birs. Rietveld-refined XRD pattern of **a** hydrated Na-bir and **b** partially dehydrated Na-bir. **c** Raman spectra of hydrated Na-bir and partially dehydrated Na-bir. TEM images of hydrated Na-bir: **d** low magnification bright field image (scale bar = 500 nm); **e**, **f** high-resolution bright field image (scale bar = 5 nm), and **g** fast Fourier transform (FFT) pattern. TEM images of partially dehydrated Na-bir: **h** low magnification bright field image (scale bar = 500 nm); **i**, **j** high-resolution bright field image (scale bar = 5 nm); **k**, **l** FFT of the regions enclosed in the red and yellow rectangles in **i**, and **m**, **n** their respective inverse FFT

The amount of interlayer crystal water in Na-bir can vary depending on its surrounding environment, leading to the changes of its interlayer distance and structure. These Na-bir structures are essentially metastable because the water contents in the structure are reversibly changed as indicated in Supplementary Fig. 1^17^. From the Thermogravimetric analysis (TGA) profiles in Supplementary Fig. 2, hydrated Na-bir is estimated to have ~8 wt% of crystal water and its Na content reaches 6 wt% based on Inductively coupled plasma-atomic emission spectroscopy (ICP-AES) analysis (Supplementary Table 1), giving the formula Na_0.27_MnO_2_·0.54H_2_O. Meanwhile, partially dehydrated Na-bir has less amount of crystal water up to 0.7 wt%, finally corresponding to the stoichiometric composition of Na_0.27_MnO_2_·0.09H_2_O. Structural details of both samples were determined by Rietveld refinement as shown in Fig. 1a, b. The layered structure of hydrated Na-bir retains the *R*-3m space group with a large *c* lattice parameter of about 21.739(6) Å. After dehydration, the main peak shifts toward higher angle, and peak broadening concurrently occurs, which is consistent with an increasing disorder in MnO_6_ slab, as well as the appearance of the spinel-like phase. While the *a* and *b* lattice parameters of both samples are quite similar, the *c* lattice parameter of the partially dehydrated sample decreases down to about 16.95(1) Å because of the reduced crystal water content and the resultant contraction of Na layer. The refinement results also demonstrate that Mn atoms occupy two different sites, 3a and 6c in partially dehydrated Na-bir but only the 3a site in hydrated Na-bir (Table 1). The migration of Mn from 3a to 6c site in partially dehydrated Na-bir results from the removal of crystal water. Oxygen and crystal water partially present in this layered structure coordinates the Mn atoms in 6c sites which are tetrahedral, thereby assembling a spinel-like phase. The occupancy of Mn at 6c (0.0, 0.0, 0.112(3)) is 0.14(1), meaning that about 28% of Mn contribute to forming the spinel-like phase in partially dehydrated Na-bir. Because the Mn atoms at the tetrahedral sites are located parallel to the oxygens, the z positions of Mn and O at 6c sites are deviated from those of conventional layered structures^18^. Raman spectroscopy (Fig. 1c) shows that hydrated Na-bir exhibits peaks at ~575–585 cm^−1^ and at ~640–650 cm^−1^ arising from the Mn–O bond stretching vibration in the basal plane of [MnO_6_] laminates. Another peak discernible at ~640–650 cm^−1^ is very prominent as the sole observable signal in partially dehydrated Na-bir, which can be attributed to the A1g mode corresponding to the Mn–O breathing vibration of tetrahedrally coordinated Mn ions^19, 20^. As shown in Fig. 1d–n, both Na-bir samples have a microflower-like morphology (Fig. 1d, h); the interlayer distances of the hydrated and partially dehydrated Na-bir were measured to be 7.27 and 5.55 Å, respectively. Fast Fourier transforms (FFT) of separate regions in the TEM image of partially dehydrated Na-bir identify both layered phases and spinel-like phases, thus demonstrating the remarkable topotactic transformation to spinel-like phases associated with the layer-to-interlayer migration of Mn ions upon dehydration. Consequently, depending on the crystal water content, Na-bir possesses either only the layered phase or both the layered and spinel-like phase.

**Table 1:** Refined structural parameters of hydrated Na-bir and partially dehydrated Na-bir

*Hydrated Na-bir*						
(*R*_wp_ = 11.6 %, *R*_p_ = 16.5, *R*_ex_ = 4.88, *S* (=*R*_wp_/*R*_ex_) = 3.38)						
Space group: *R*-3m, *a* = *b* = 2.8510(7) Å, *c* = 21.739(6) Å, Vol = 153.03(7) Å^3^, *α* = *β* = 90°, *γ* = 120°						
**Atom**	**Site**	***x***	***y***	***z***	***B***_iso_ (Å^2^)	**Occ.**
Na	3b	0.0	0.0	0.5	0.7(2)	0.135
Mn	3a	0.0	0.0	0.0	0.7(2)	0.5
O	6c	0.0	0.0	0.374(2)	0.7(2)	1
*Partially dehydrated Na-bir*						
(*R*_wp_ = 10.0%, *R*_p_ = 6.77, *R*_ex_ = 3.98, *S* (=*R*_wp_/*R*_ex_) = 2.51)						
Space group: *R*-3m, *a* = *b* = 2.8408(6) Å, c = 16.95(1) Å, Vol = 118.5(1) Å^3^, *α* = *β* = 90°, *γ* = 120°						
**Atom**	**Site**	***x***	***y***	***z***	***B***_iso_ (Å^2^)	**Occ.**
Na	3b	0.0	0.0	0.5	2.0(3)	0.135
Mn1	3a	0.0	0.0	0.0	2.0(3)	0.35(1)
Mn2	6c	0.0	0.0	0.112(3)	2.0(3)	0.14(1)
O	6c	0.0	0.0	0.394(2)	2.0(3)	1

### Electrochemical characterization

**Fig. 2:**
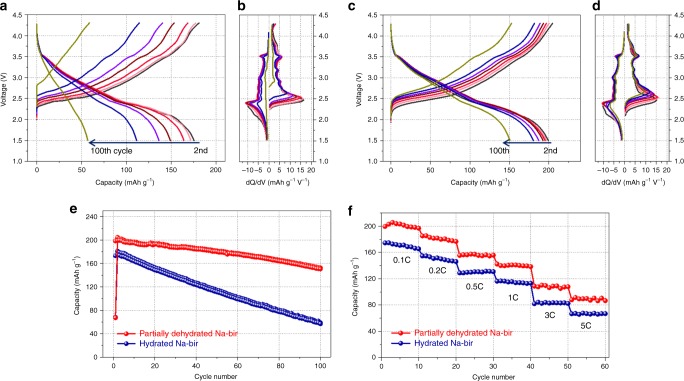
Electrochemical performances of hydrated and partially dehydrated Na-birs. Charge/discharge profiles and the differential capacity vs. voltage plots (d*Q*/d*V*) of **a**, **b** hydrated and **c**, **d** partially dehydrated Na-bir at the 2nd, 5th, 10th, 20th, 30th, 50th, and 100th cycle. **e** Cyclic retention at 0.1 C and **f** discharge capacities of hydrated and partially dehydrated Na-bir at various rates from 0.1 to 5 C

The effects of structural rearrangement via partial dehydration on electrochemical properties are presented in Fig. 2. The first charge curve demonstrates that both hydrated and partially dehydrated Na-bir have similar charge capacities corresponding to ~0.275 mol of Na ions, as shown in Supplementary Fig. 3a. However, the partially dehydrated Na-bir displays much higher first discharge capacity than the hydrated Na-bir. This increased capability seems to originate from the activation of Na-occupying sites induced by the structural rearrangement. This inference is clearly confirmed in Supplementary Fig. 3b, where, in the absence of an initial charge, the partially dehydrated Na-bir can accommodate additional ~0.1 mol Na ions compared to its hydrated counterpart. Figure 2a–d show the charge/discharge profiles of Na-birs at the 2nd, 5th, 10th, 20th, 30th, 50th, and 100th cycles at 0.1 C between 1.5 and 4.3 V and the corresponding differential capacity vs. voltage plots (d*Q*/d*V*). An anodic peak at ~2.5 V is coupled with the corresponding cathodic peak at ~2.4 V, which can be attributed to the redox reaction of Mn^3+/4+^. Above 3.0 V, the peaks related to complex structural changes can be observed, although they remain more apparent up to the 100th cycle in the partially dehydrated Na-bir. These results indicate that the partially dehydrated Na-bir showed a high electrochemical reversibility and, correspondingly, exhibited a much improved cyclic retention up to 100 cycles (Fig. 2e). Moreover, the partially dehydrated Na-bir retained a discharge capacity of 150 mAh g^−1^ after 100 cycles, corresponding to 75% of its initial discharge capacity, while the hydrated Na-bir retained only 32% of its initial capacity after 100 cycles. The improved cycling stability of partially dehydrated Na-bir is quite impressive because high capacity at higher voltage range induced fast degradation in conventional layered materials. Furthermore, the kinetic advantage of partially dehydrated Na-bir is also demonstrated by the rate capability test (Fig. 2f). When the partially dehydrated Na-bir is charged and discharged at 5 C, it could still exhibit 87.96 mAh g^−1^, which corresponds to 44% of the discharge capacity at 0.1 C. To further investigate the kinetic properties of Na-bir, Na^+^ diffusion coefficients are obtained from galvanostatic intermittent titration technique (GITT) (Supplementary Fig. 4 and Supplementary Note 1). It was very clear that controlling the crystal water contents can facilitate the phase transformation from layered to spinel-like structure, which provides higher Na^+^ diffusion environment in the structure. Therefore, the role of crystal water contents looks critical for the electrochemical performances of Na-bir. To make sure this point, we also analyzed the electrochemical performances of fully dehydrated Na-bir which was heat-treated at 300^o^C for 5 h. Interestingly, as Nam et al. reported^9^, the fully dehydrated Na-bir shows the most inferior electrochemical performances among the samples underscoring the importance of proper contents of crystal water in the structure (Supplementary Figs. 5, 6 and Supplementary Note 2).

### Reversible structural change between layered and spinel-like phases

**Fig. 3:**
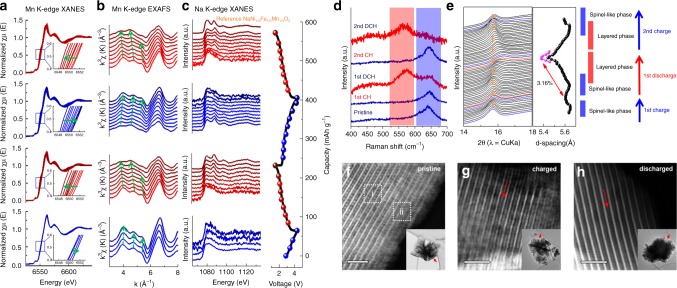
Reversible structural changes of partially dehydrated Na-bir during the cycling. **a** Ex situ Mn K-edge XANES spectra, **b** Ex situ *k*^3^-weighted Mn K-edge EXAFS spectra of partially dehydrated Na-bir during the initial two cycles. **c** Ex situ Na K-edge XANES spectra of partially dehydrated Na-bir during the initial 2 cycles. **d** Ex situ Raman spectra of partially dehydrated Na-bir in the pristine, 1st charge, 1st discharge, 2nd charge, and 2nd discharge state. **e** In situ XRD patterns of partially dehydrated Na-bir during Na ion insertion/extraction at a current density of 30 mA g^−1^. High-resolution ADF-STEM images of **f** pristine, **g** charged, and **h** discharged partially dehydrated Na-bir. The scale bars are 2 nm

Inspired by the reversible structural change and the improved electrochemical performances, we investigated the reaction mechanism during Na ion insertion/extraction in partially dehydrated Na-bir (Fig. 3). In Fig. 3a and Supplementary Fig. 7, the normalized Mn K-edge X-ray absorption near edge structure (XANES) spectra shift towards higher energies during Na ion extraction and move back to the original position during Na ion insertion. The edge positions of Mn K-edge XANES spectra after first charge and discharge are identical with those after second charge and discharge, respectively, implying that the redox process is extremely reversible. It has been reported that the *k*^3^-weighted EXAFS spectra can identify the characteristic connectivity of MnO_6_ octahedra composed of edge-sharing di-μ-oxo bridging between Mn^4+^ ions, corner-sharing mono-μ-oxo bridging between Mn^3+/4+^ ions, and combination of both in various MnO_*x*_ compounds. In Extended X-ray absorption fine structure (EXAFS), spectral similarity between the investigated compound and the reference compounds is indicative of identical local structure^21–24^. Hence, this technique is suitable for substantiating our aforementioned structural model that eliminating crystal water from the interlayer is accompanied by the migration of Mn^3+^ ions into the interlayer vacancy, leading to a spinel-like phase with edge-sharing and corner-sharing MnO_6_ octahedra. Compared with the previously reported Mn K-edge EXAFS spectra for MnO_*x*_ compounds, the spectra for the partially dehydrated Na-bir in pristine and desodiated states clearly indicate both edge and corner-sharing MnO_6_ octahedra with triple-hump peak in 3–6 Å^−1^ region (Fig. 3b). After Na ion was inserted back into the structure, the EXAFS oscillations are indicative of its structure transformation into a layered phase with edge-sharing MnO_6_ octahedra^21, 25^. In the *k*^3^-weighted EXAFS spectra at different states of charge and discharge, the distinguishable EXAFS oscillations change reversibly between a double-hump and a triple-hump peak in the 3–6 Å^−1^ range during electrochemical cycling, which means reversible structural changes between layered and spinel-like phases during sodiation/desodiation (Supplementary Fig. 8). In this context, the theoretical calculation represents the reduced electronic configuration toward Mn^3+^ valence state at the tetrahedral site (migrated state) comparing to the Mn^4+^ valence state at the octahedral site (original state) in the projected (partial) density of states (PDOS) of Mn *d*-orbital and Bader charge analysis as shown in Supplementary Fig. 9 and Supplementary Note 3.

The Na K-edge spectra of partially dehydrated Na-bir at fully discharged state is very similar to that of the reference layered compound, NaNi_1/3_Fe_1/3_Mn_1/3_O_2_, which exhibits two major characteristic peaks at ~1080 and ~1087 eV as shown in Fig. 3c and Supplementary Fig. 10, suggesting that its Na local environment at fully discharged state is similar to that of the typical layered compound. Because the reversible movement of Mn ions between MnO_2_ layer (3a) and the interlayer (6c) induces the phase transformation between the layered and spinel-like structure, all the Na ions look like being octahedrally coordinated by the neighboring oxygen atoms at the discharged state with the layered structure. By contrast, at the charged state having the spinel-like structure, the Na ions are partially coordinated by four neighboring oxygen atoms in the tetrahedral sites. The latter can be modeled by first-principles atomistic relaxation for the Mn-migrated structure as shown in Supplementary Fig. 11. The investigation on Mn and Na local environments by XAS clearly proves high reversibility of the local structural change as the material transforms between the layered and spinel-like phases during electrochemical cycling. This reversible phase transformation has also been demonstrated by ex situ Raman spectroscopy (Fig. 3d). The characteristic Raman band of partially dehydrated Na-bir is changed after charge, which results from an increased separation between two successive slabs due to the strengthened repulsion between the oxygen atoms from the adjacent slabs. As mentioned above, the sharp band at around 640–650 cm^−1^ is typically assigned to tetrahedrally coordinated Mn–O bonding, which is indicative of a spinel-like phase. The Raman spectra of partially dehydrated Na-bir show a newly emerged peak at ~575–585 cm^−1^ together with the decrease of band intensity at ~640–650 cm^−1^ after discharge, which implies that it has an arrangement of layered structure. As well consistent to the aforementioned analysis results, the partially dehydrated Na-bir shows high reversibility^20^. Therefore, controlling crystal water contents in birnessite looks critical to enable the structural reversibility because hydrated Na-bir exhibited a clearly limited reversibility as shown in Supplementary Fig. 12 and Supplementary Note 4.

The in situ XRD patterns and calculated variation of *d*-spacing for the partially dehydrated Na-bir during electrochemical cycling are shown in Fig. 3e. Because of the large interlayer distance of partially dehydrated Na-bir, the main Bragg peak representing the interlayer distance is positioned at a low angle below 16°. At the initial state of charge, this Bragg peak is shifted to a higher angle which is attributable to triple corner-sharing Mn or the migration of MnO_6_ octahedra. During discharge, Na ion is intercalated into the spinel-like phase accompanied by a slight increase in *d*-spacing. At the depth of discharge of ~30%, this Bragg peak begins to shift to higher angles due to the structural transformation from spinel-like to layered structure. Na ion insertion into the layered structure induced the contraction of interlayer distance by decreasing the oxygen repulsion. Consequently, a new peak of hexagonal layered structure at a high angle emerges, with a total *d*-spacing variation of 3.16% at the fully charged state. Up to a state of charge of ~70%, Na ion extraction from the layered structure of partially dehydrated Na-bir is accompanied by an expanded interlayer distance due to the increased oxygen repulsion between MnO_6_ octahedra. After being fully charged during 2nd cycle, the partially dehydrated Na-bir has reverted completely to the spinel-like phase, resultantly demonstrating the full reversibility of the phase transformation. The phase transformation by reversible Mn migration is directly observed by ADF-STEM analysis. For the pristine sample, it is revealed that the spinel-like structure (denoted by the box i) coexists with the layered structure (denoted by box ii) due to the Mn migration into the Na layer during the partial dehydration (Fig. 3f). After charge, the desodiation induces the phase transformation to the spinel-like structure in which Mn ions occupy the tetrahedrally coordinated sites by oxygen in the interlayer; the brighter contrast in the interlayer corresponds to this structural feature (denoted by red arrow in Fig. 3g) due to the stronger scattering signal of Mn than that of Na. After discharge, very weak contrast in the interlayer is observed (denoted by red arrow in Fig. 3h). These extra STEM/EDX analysis results (Supplementary Fig. 13) corroborate that the Mn ions in the interlayer are reversibly migrated back to octahedral sites in the transition metal layers during discharge. Altogether, these ex situ and in situ analyses prove the completely reversible phase transformation between the layered and spinel-like structure in partially dehydrated Na-bir during Na ion insertion/extraction. Furthermore, this unique phenomenon is beneficial for its improved cyclic stability and facilitated Na ion diffusivity, which may arise from well-relieved mechanical stress and strain induced by the removal of crystal water and the following structural rearrangement as demonstrated by ex situ analyses including SEM, XPS, ICP-AES, XRD, and TGA after 100 cycles (Supplementary Figs. 14–17, Supplementary Table 2 and Supplementary Note 5–9). Therefore, these results stand in contrast to the conventional paradigm of layered materials, where most of phase transformations including layered-to-spinel and so on are irreversible that need to be suppressed at the expense of limited electrochemical performances. Even if the chronic problems like Mn dissolution, electrolyte decomposition, etc. look much more dominant with hydrated Na-bir, these seem to still affect the partially dehydrated Na-bir badly. So, a search for proper electrolyte in this regime of electrode material may be required to make this breakthrough result more valuable performance-wise.

### First-principles calculations of Mn ion migration barrier

**Fig. 4:**
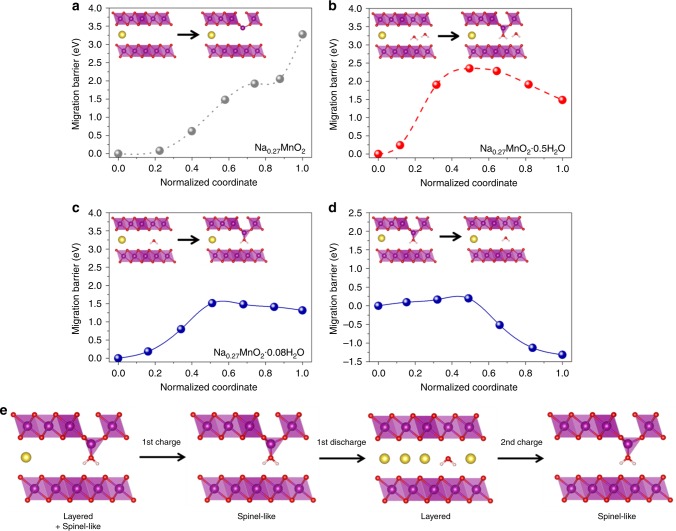
Calculations of Mn ion migration barriers depending on the contents of crystal water. Migration barriers of Mn ions from octahedron in Mn layer to tetrahedron in Na layer in **a** Na_0.27_MnO_2_, **b** Na_0.27_MnO_2_·0.5H_2_O, **c** Na_0.27_MnO_2_·0.08H_2_O, and **d** Migration barrier for the reverse process in Na_0.27_MnO_2_·0.08H_2_O. **e** Schematic illustration for the reversible structural changes of partially dehydrated Na-bir during Na ion insertion/extraction

In order to unveil the mechanism for the reversible phase transformation between layered and spinel-like structure, we utilized first-principles calculations for the migration barrier of Mn ions from an octahedral geometry in MnO_6_ layer to a tetrahedral geometry in Na layer^1, 2^. First of all, it was impossible to theoretically incorporate Na ions into the spinel structure by assuming that Na ions occupy the alkali ion sites of Li-based spinel structures, due to the larger *d*-spacing of sodium-incorporating materials. Hence, it is difficult for Na-bir without any crystal water (similar to O3-Na_*x*_MnO_2_) to form a stable tetrahedral geometry even if Mn ions migrate to the Na layer as shown in Fig. 4a. Because of the wide slab-to-slab separation, the tetrahedral Mn fails to share any oxygen with adjacent MnO_6_ layer and the migration energy reaches ~3.3 eV, meaning that the spinel-like structure is difficult to be formed in Na-bir without crystal water. Herein, our computational model was based on O3-Na_0.25_MnO_2_, consisting of 3 Na, 12 Mn, and 24 O atoms. For Na-bir with crystal water, we assumed the Na-birs with 0.5 and 0.08 mol crystal waters, which each correspond to two crystal waters in every single Na layer and one crystal water in every 3 Na layers to investigate the dependency of migration energy on the contents of crystal water in the Na layer, as shown in Fig. 4b, c. These all Na-birs showed lower migration energy than the crystal water-free Na-bir, implying that tetrahedral Mn is coordinated by the oxygen of crystal water instead of that of neighboring MnO_6_ slab and thus the reversible phase transformation of Na-bir is closely associated with the metastable spinel-like phase, the formation of which is facilitated by the interaction between Mn ions and crystal water. However, a relatively higher migration barrier (~2.35 eV) of Mn ions was obtained for Na-bir with 0.5 mol of H_2_O (Fig. 4b), where too high content of crystal water seems to interfere with and hinder the formation of the spinel-like phase. On the other hand, Na-bir with 0.08 mol of H_2_O has a lower migration barrier (~1.5 eV), which enables the formation of spinel-like structure as represented in Fig. 4c, d. To determine the optimum amount of crystal water for this reversible phase transformation, we additionally calculated the migration energy barrier of Mn ion in Na-birs with 0.17 and 0.25 mol of crystal waters as shown in Supplementary Fig. 18. Both of Na-birs also allowed the migration of Mn ions to the Na layer in the same way as that with 0.08 mol of crystal water. Due to the large separation between MnO_6_ slabs in Na-bir, the interlayer interactions of both Na birs tend to commonly weaken during the migration of Mn ion in the same way as for the Na-bir with 0.08 mol of crystal water; in all three structures, their Mn migration energies just reach a similar value of about 1.5 eV. Recent study on Mn migration in birnessite structures reported by Yang et al. also indicates that the crystal water mitigates the higher migration barrier of Mn ion and facilitate the spinel-to-layered phase transformation^26^. Hence, it is apparent that an appropriate amount of crystal water can facilitate the formation of the spinel-like phase while preventing the irreversible formation of the stable spinel phase (MnO_6_) where oxygen ions from adjacent MnO_6_ slabs coordinate the tetrahedral Mn. This reversible phase transformation between the layered and spinel-like structure of partially dehydrated Na-bir are schematically illustrated in Fig. 4e based on experimental and first-principle calculation results. In addition, we explore whether it is possible for Mn ions to migrate to the location of Na ions in Na layer from the tetrahedral site formed with crystal water. As shown in Supplementary Fig. 19, the migrated Mn ion could not generate an octahedron with adjacent oxygen ions due to the elongated *d*-spacing between Mn layers from the co-existence of crystal water and Na ion. This indicates that Mn ion in the original octahedral site of Mn layer is likely to migrate to the tetrahedral site formed by the crystal water, but it is hard to migrate to the octahedral site in Na layer, underscoring the reversible layered-to-spinel phase transformation observed in this work. Also, the formation of spinel-like phase helps to attain higher stability by preventing the extraction of crystal water. Supplementary Fig. 20, Supplementary Table 3, and Supplementary Notes 10, 11 demonstrate that the extraction energy of crystal water between Mn layers is much lower than that of the crystal water bonded to the migrated Mn ions underscoring that the crystal water in partially dehydrated Na-bir is much more stable than that in hydrated Na-bir. This observation again supports the reason why partially dehydrated Na-bir can present high stability and capacity retention after 100 cycle.

## Discussion

We investigated an unprecedented reversible phase transformation between layered and spinel-like structure during reduction/oxidation of a layered-structure oxide. This surprising result was observed in the birnessite structure. The mechanism for its phase reversibility was elucidated by in-depth structural characterizations combined with first-principles calculations. These results indicate the formation of a metastable spinel-like phase during ion extraction, which enables this unusual reversibility in its phase transformation. Moreover, the migration energy of Mn ions from an octahedral geometry in MnO_6_ slab to a tetrahedral geometry in Na layer turned out to be regulated by the existence of crystal water in Na layer. The findings from the first-principles calculation coupled with advanced structural analyses substantiated that the contents of crystal water are very important in forming the metastable spinel-like structure which plays a key role in high reversibility of phase transformation. Furthermore, this reversible transformation was accompanied by the successful accommodation of volume change enabling very stable cyclic retention. We firmly believe that these unprecedented findings provide invaluable insight into the search for novel class of electrode or electronic materials that are away from the limitation given by their characteristic crystal structures. Hence, this work will not only broaden the structural candidates as electrode materials in advanced secondary batteries, but also establish new research directions in materials science.

## Methods

### Synthesis of hydrated Na-bir, partially dehydrated Na-bir and fully dehydrated Na-bir

Hydrated Na-bir was synthesized in an aqueous solution using a CEM Discover microwave accelerated reactor system (MARS 5). Concentrated NaMnO_4_ (40 wt% in H_2_O) was purchased from Sigma-Aldrich and used without further purification. In a typical procedure, the NaMnO_4_ solution (2.5 mL) was added to deionized water (40 mL) in a 100 mL microwave reaction vessel. The solution was then heated to 220 °C for 6 h under the sealed vessel mode with microwave irradiation. This microwave reaction was conducted in air atmosphere. The resulting brownish-black solid product was isolated by centrifugation, washed with deionized water three times, and dried at 60 °C in a vacuum oven overnight. To obtain the partially dehydrated Na-bir and fully dehydrated Na-bir, the brownish-black solid product was each heat-treated at 170 and 300 ^o^C for 5 h with a temperature ramp of 5 °C min^−1^ to remove the crystal water between MnO_6_ slabs. The synthesized samples were sealed to avoid atmospheric moisture before use.

### Characterizations

The XRD patterns of hydrated Na-bir and dehydrated Na-bir powder were collected on the 9B HRPD beamline at Pohang Light Source-II (PLS-II) in South Korea. The incident X-ray was vertically collimated using a mirror and monochromatized to a wavelength of 1.4865 Å using a double-crystal Si(111) monochromator. The diffraction patterns were collected in an angular 2*θ* range of 10–130° with a step size of 0.02°, while rotating the sample to minimize the preferred orientation effect. The HRPD patterns were calculated using the Fullprof program package to obtain the structural details.

The morphologies of the hydrated Na-bir and dehydrated Na-bir were characterized by field-effect scanning electron microscopy (FE-SEM; JEOL JSM-6700F, operated at 10 and 30 kV), and high-resolution-transmission electron microscopy (HR-TEM, FEI Tecnai G2 F30, operated at 300 kV), using the Gatan Digital Micrograph program. Annular dark field (ADF) imaging mode was employed to observe the structures of the partially dehydrated Na-bir samples on an aberration-corrected scanning transmission electron microscope (STEM, JEM-ARM200CF, JEOL) equipped with a cold-field emission electron gun at an accelerating voltage of 80 kV. The detector angle range for the ADF imaging was ~45–180 mrad and the probe forming angle was ~23 mrad. Experimental ADF-STEM images were denoised by the Wiener filtering method implemented in a commercial software program (HREM Filter Pro, HREM research Ltd.). Energy-dispersive X-ray mapping for the Na-bir samples before and after charging was performed on the same ADF-STEM imaging mode with a dual-type silicon drift detector (JED-2300T, JEOL Ltd.) providing a large effective solid angle (~1.2 sr). The residual moisture content in the products was determined by thermogravimetric analysis (TGA). The compositions and concentration of Mn^2+^ deposited on Na anode were determined by ICP-AES (OPTIMA 7300 DV, Perkin-Elmer). The XPS spectra were analyzed by K-Alpha+, ThermoScientific installed at Hanyang Center for Research Facilities (Seoul). The Mn K-edge XAFS was conducted under the transmission mode at the 8C (Nano XAFS) beamline in the Pohang Light Source-II (PLS-II). The soft X-ray absorption spectra were obtained at beamline 10D XAS KIST (Korea Institute of Science and Technology) in the PLS-II. The Na K-edge XANES were collected under total electron yield mode in vacuum ~5 × 10^–8^ Torr. The in situ XRD measurements of partially dehydrated Na-bir was performed at beamline 9A (U-SAXS) PLS-II using a ccd image plate detector with wavelength of 0.62147 Å and beamline 3D-XRS PLS-II using a MAR3450 image plate detector with wavelength of 1.03337 Å in the transmission mode. The storage ring was operated at electron energy of 3.0 GeV and a current of 320 mA. The X-ray exposure time was 10 s, and it took about 3 min to get a single pattern. The two-theta angles of all the patterns presented have been recalculated to the corresponding angles for *λ* = 1.54 Å, which is the wavelength of conventional X-ray tube source with CuKα radiations, for an easy comparison with other published results.

### Electrochemical measurements

Hydrated Na-bir and partially dehydrated Na-bir electrodes were prepared from slurries with the following composition: active material (80 wt%); acetylene black (10 wt%), which was used as a conductive agent, and a binder (polyvinylidenedifluoride, 10 wt%), which was dissolved in N-methyl-2-pyrrolidone (NMP). This slurry was pasted onto an aluminum-foil current collector and dried at 70 °C for 5 h under vacuum (10^−3^ Torr). Electrodes with a diameter of 12 mm were punched, and the average active material loading density of hydrated Na-bir and dehydrated Na-bir electrodes corresponded to 1.91 mg cm^−2^. The electrochemical properties of the prepared electrodes were evaluated using CR2032 coin-type cells assembled in an Ar-filled glove box. Na metal was used as a counter electrode, and a solution of NaPF_6_ (1 M) in ethylene carbonate (EC) and diethyl carbonate (DEC) (1:1, v/v) was employed as the electrolyte. Charge/discharge tests were performed in a range of 1.5–4.3 V (vs. Na/Na^+^) at 0.1 C. A rate of 1 C corresponded to about 200 mA g^−1^. The GITT measurement was performed at an electrochemical workstation (WonATech, WBCS3000) by applying a constant current flux (20 mA g^−1^) for 12 min, followed by open-circuit equilibration time for 2.5 h. Diffusion coefficient values are calculated from GITT curve by using the following equation:

1 $${\tilde D} = \frac{4}{{\pi \tau }}\left( {\frac{{m_{\mathrm{{B}}}V_{\mathrm{{M}}}}}{{M_{\mathrm{{B}}}S}}} \right)^2\left( {\frac{{\Delta E_{\mathrm{{s}}}}}{\Delta{E_{\mathrm{{t}}}}}} \right)^2\quad \left( {\tau \ll \frac{{L^2}}{{{\tilde D}}}} \right)$$

where ${\tilde D}$ is the chemical diffusion coefficient, *m*_B_ is the mass of active material, *M*_B_ is the atomic weight, *V*_M_ is the molar volume, *S* is the surface area of the electrode, Δ*E*_t_ is the total change of voltage during the discharge at constant current, and Δ*E*_s_ is the change of the steady-state voltage. For in situ XRD measurements, the prepared electrode was incorporated into coin cells with a Kapton window.

### Calculation details

First-principles calculations for structural relaxations and atomic migrations of Na-bir were investigated by spin-polarized density functional theory (DFT) with the projector augmented-wave (PAW) method using Vienna ab initio simulation package (VASP)^27, 28^. The Perdew–Burke–Ernzerhof (PBE) of generalized-gradient approximation (GGA) was utilized for the exchange-correlation functional^29^. The Hubbard *U* correction was used for reflecting the strong correlation of 3*d* electrons, and the *U* value of 3.9 eV for Mn ion were adopted from the previous study for Na layered oxide^30^. From the convergence test, a plane-wave cut-off energy of 550 eV was used and *k*-point mesh of 4 × 4 × 2 was sampled. The climbing-image nudged elastic band (ci-NEB) method^31^ was utilized to calculate the migration barriers of Mn ions in Na-bir structures. We used Bader charge analysis to estimate the electronic charge transfer^32^. The calculation model was established based on the structural characterization, such as Rietveld refinement, TGA, and ICP-AES. The hydrated Na-bir was modeled by Na_0.25_MnO_2_·0.5H_2_O of *R*-3m space group consisting of three Na, 12 Mn, and 24 O ions with 6 water molecules, and the partially dehydrated structure was represented by Na_0.25_MnO_2_·0.08H_2_O of *R*-3m space group consisting of three Na, 12 Mn, and 24 O ions with one water molecules. Relaxed *d*-spacing between Mn layers without crystal water is 5.8539 Å, and the *d*-spacing increases to 6.4506 Å when a crystal water remains between Mn layers.

## Supplementary information

Supplementary Information

## Data Availability

The data that support the findings of this study are available from the corresponding author upon request.
