# Supplementary material for: Triggered reversible phase transformation between layered and spinel structure in manganese-based layered compounds
Source: Nat Commun. 2019 Sep 2;10:3385. doi: 10.1038/s41467-019-11195-9 (PMC6718664; doi:10.1038/s41467-019-11195-9)
Supplement: Supplementary file 1 — Supplementary Information [file 41467_2019_11195_MOESM1_ESM.pdf]

# **Supplementary Information** for

## Triggered Reversible Phase Transformation between Layered and Spinel Structure in Manganese-based Layered Compounds

Jo *et al.*

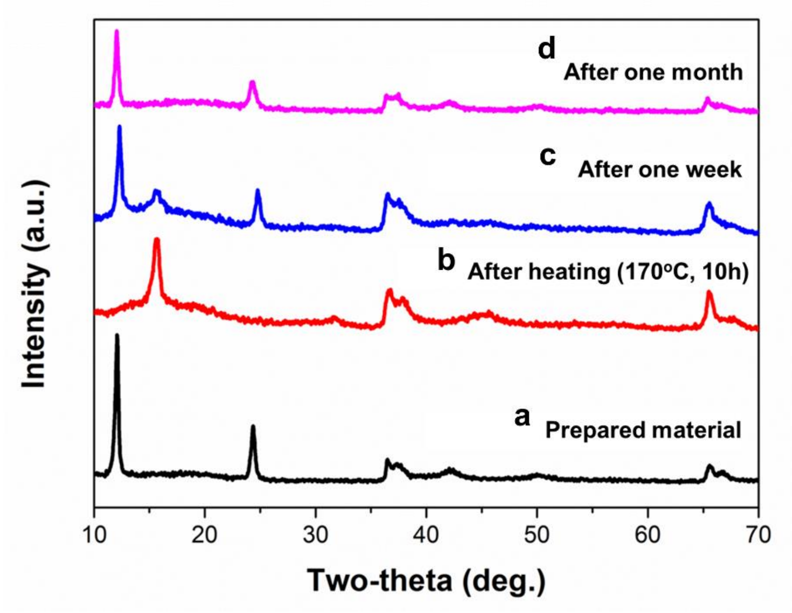


**Supplementary Figure 1** X-ray diffraction patterns of the Na-bir (a) as prepared material, (b) after heating to 170°C for 10h, reabsorption of atmospheric moisture (c) after one week and (d) one month.

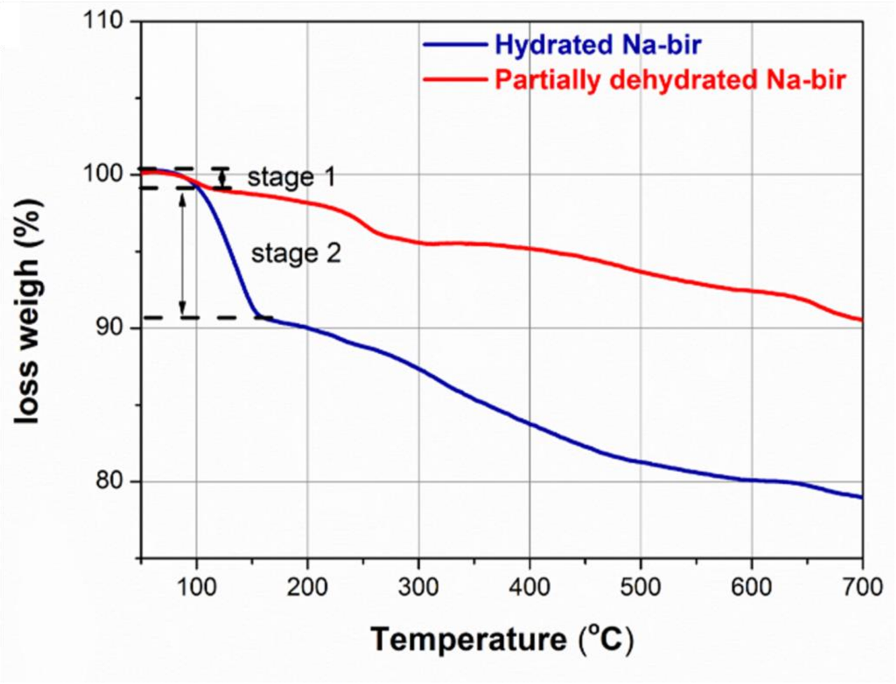


**Supplementary Figure 2** TGA curves of the hydrated and partially dehydrated Na-bir samples.

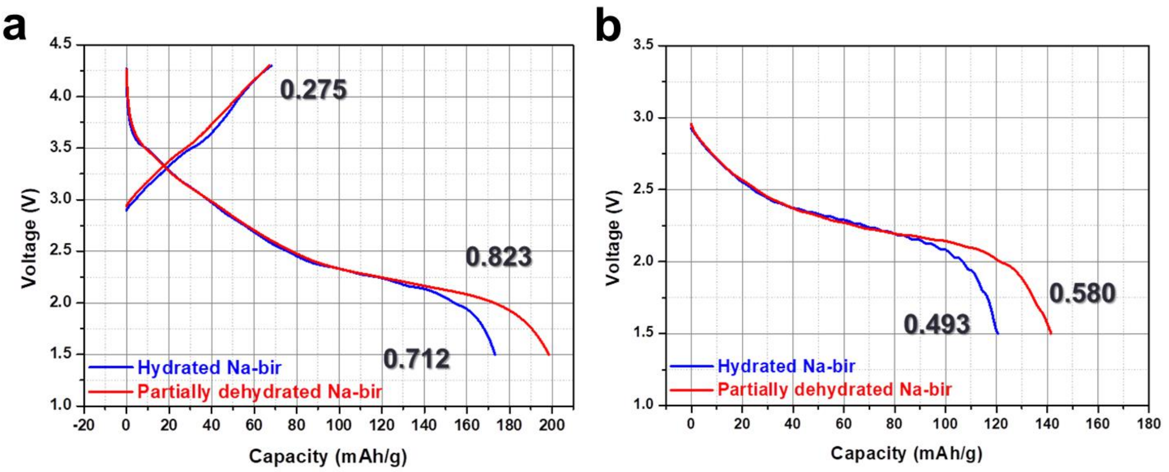


**Supplementary Figure 3 Electrochemical behavior of hydrated and partially dehydrated Na-birs.** (a) Galvanostatic charge-discharge curves and (b) initial discharge curves of hydrated and partially dehydrated Na-bir without an initial charge step.

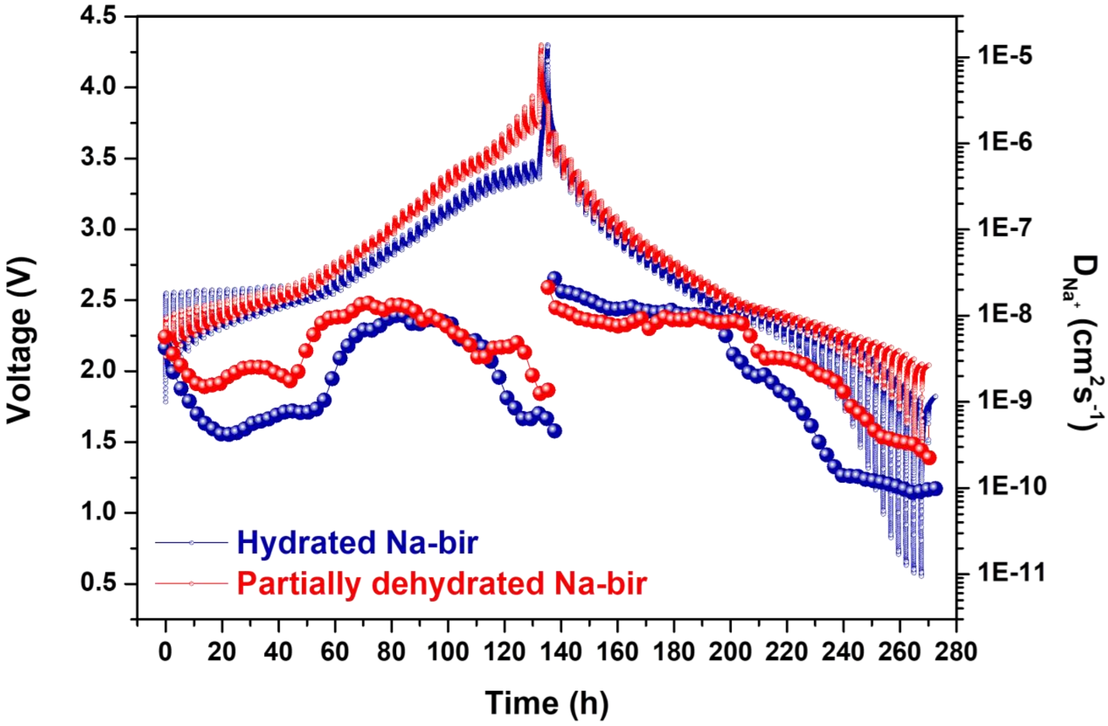


**Supplementary Figure 4** GITT curve and the calculated diffusion coefficient of hydrated Na-bir and partially dehydrated Na-bir acquired using a current flux of 20 mA $g^{-1}$ for 12 min while the relaxation time of 2.5 h.

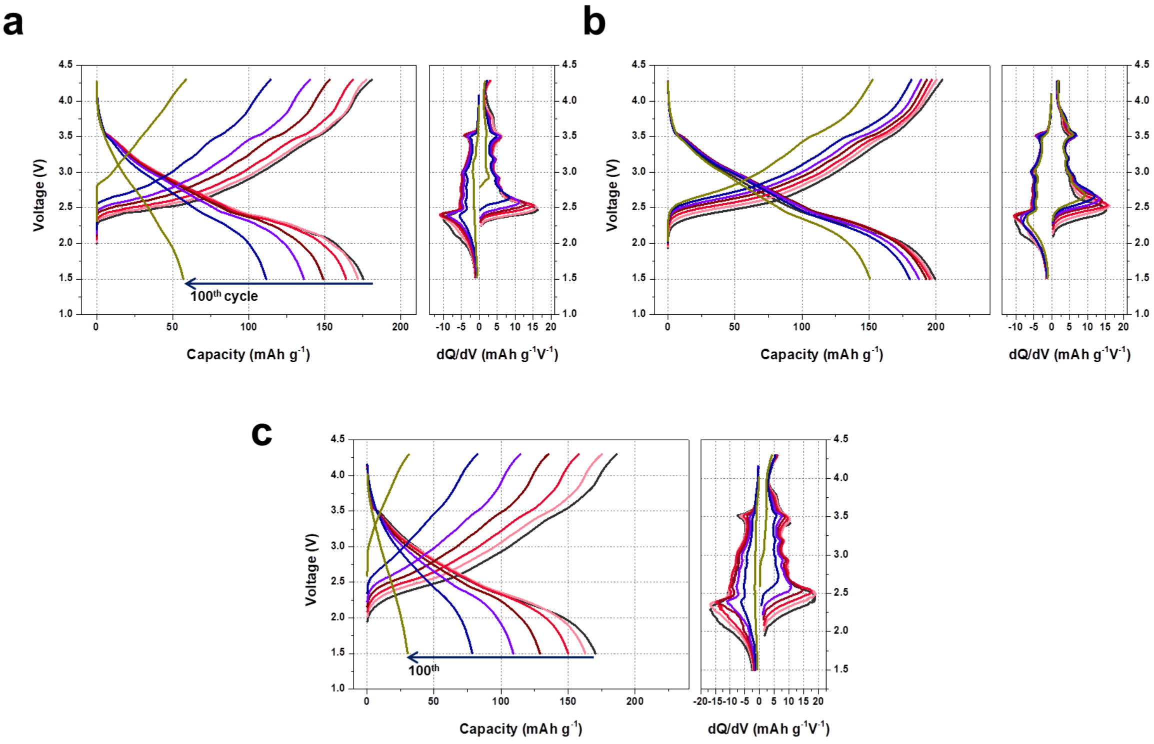


**Supplementary Figure 5 Electrochemical performances of hydrated, partially dehydrated, and fully dehydrated Na-birs during 100 cycles.** Charge/discharge profiles and the differential capacity vs. voltage plots (dQ/dV) of hydrated, partially dehydrated Na-bir and fully dehydrated Na-bir at the 2$^{nd}$, 5$^{th}$, 10$^{th}$, 20$^{th}$, 30$^{th}$ 50$^{th}$, and 100$^{th}$ cycle.

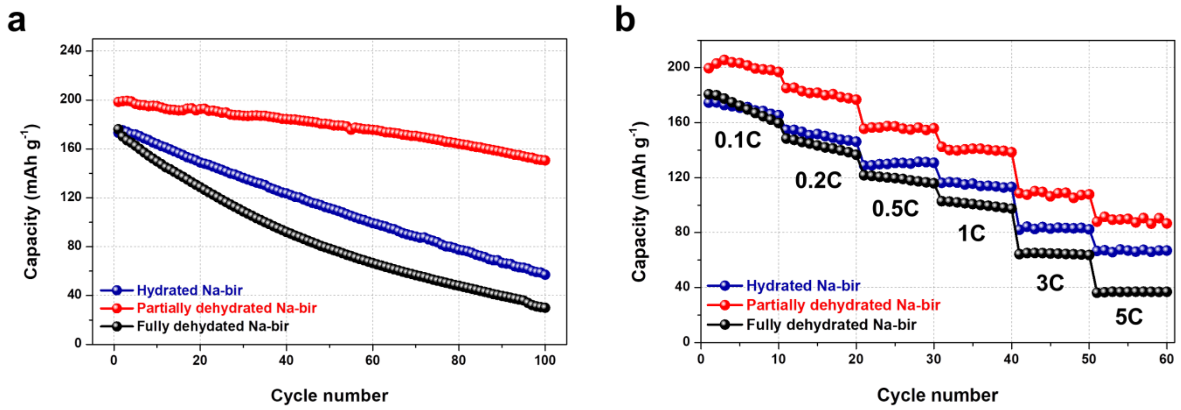


**Supplementary Figure 6 Electrochemical performances of hydrated, partially dehydrated, and fully dehydrated Na-birs.** (a) Cyclic retention at 0.1C and (b) discharge capacities of hydrated, partially dehydrated and fully dehydrated Na-bir at various rates from 0.1C to 5C.

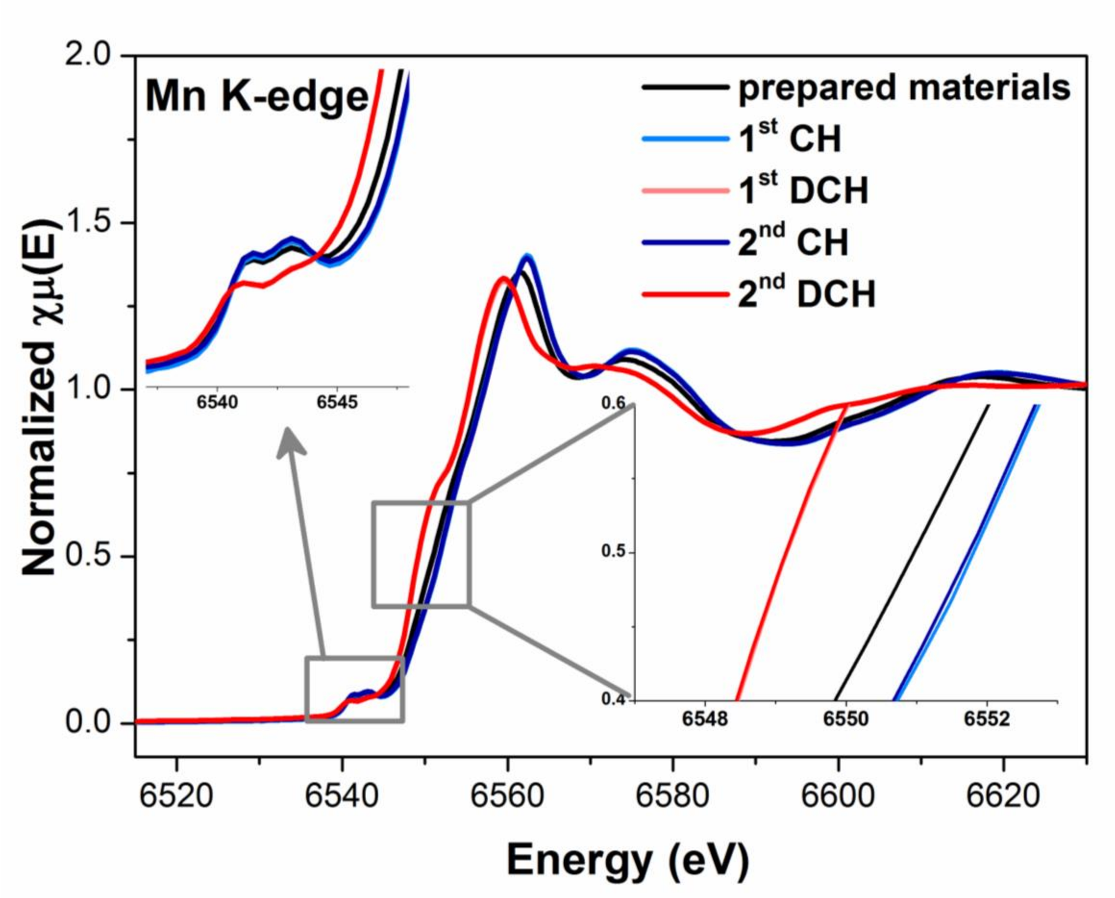


**Supplementary Figure 7** Selected XANES spectra of partially dehydrated Na-bir at pristine, 1$^{st}$ charge, 1$^{st}$ discharge, 2$^{nd}$ charge, and 2$^{nd}$ discharge states.

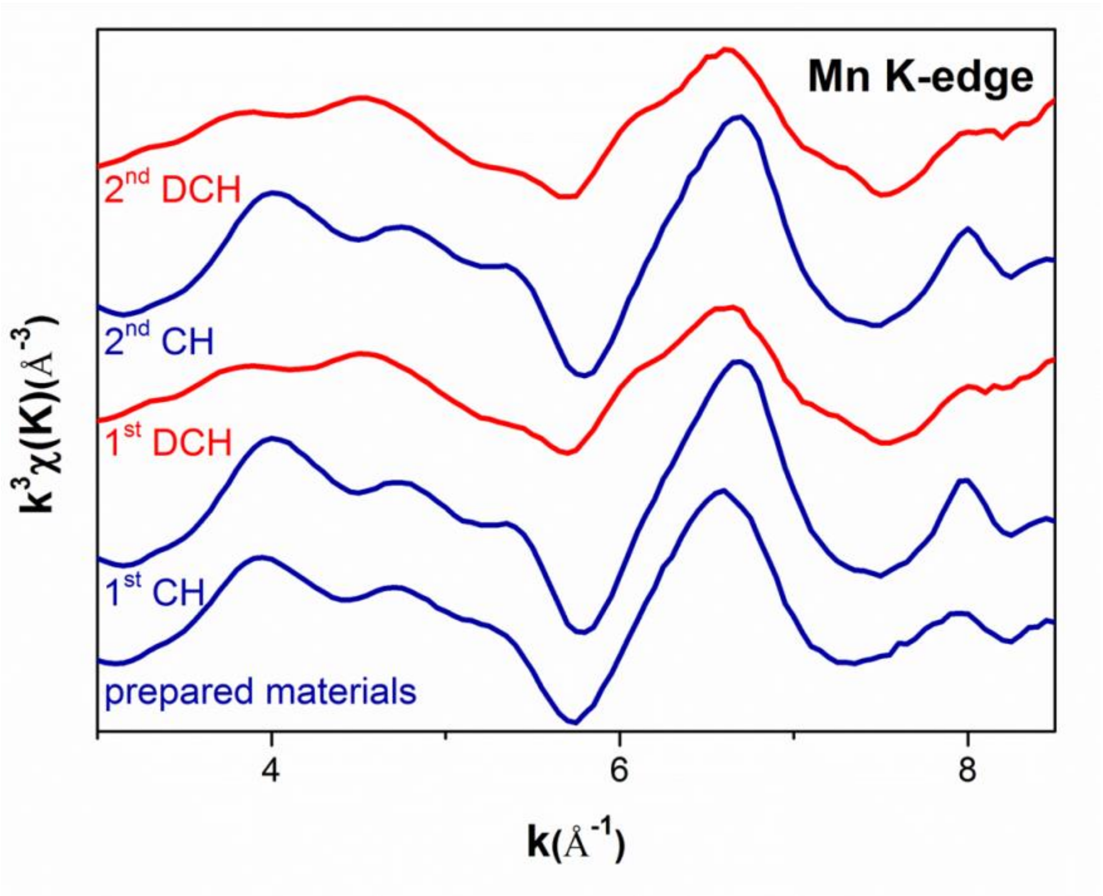


**Supplementary Figure 8** Selected $k^3$-weighted Mn K-edge EXAFS spectra of partially dehydrated Na-bir at the pristine, 1st charge, 1st discharge, 2nd charge and 2nd discharge.

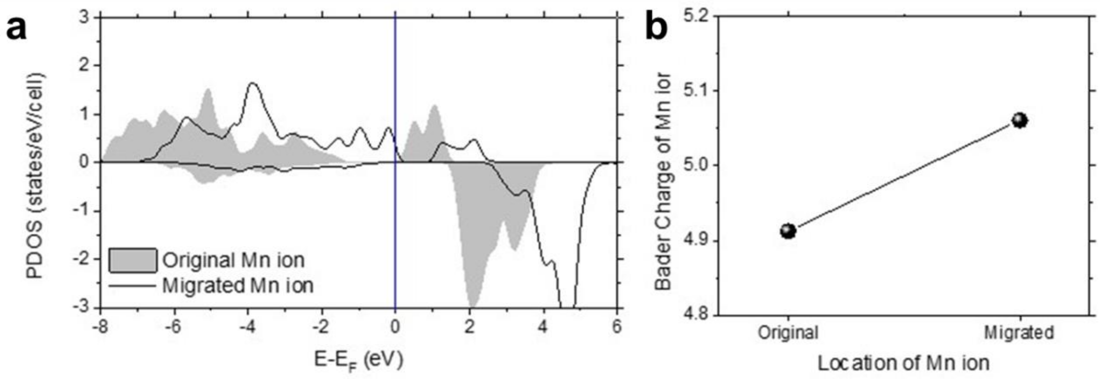


**Supplementary Figure 9 Calculations of projected density of states and Bader charge.** (a) Projected (partial) density of states (PDOS) of d-orbital in the original Mn ion at the octahedral site (gray filled area) and the migrated Mn ion at the tetrahedral site (black solid line). (b) The corresponding Bader charge of Mn ions at the original and migrated sites. The fermi level ($E_F$) set to zero (blue solid line).

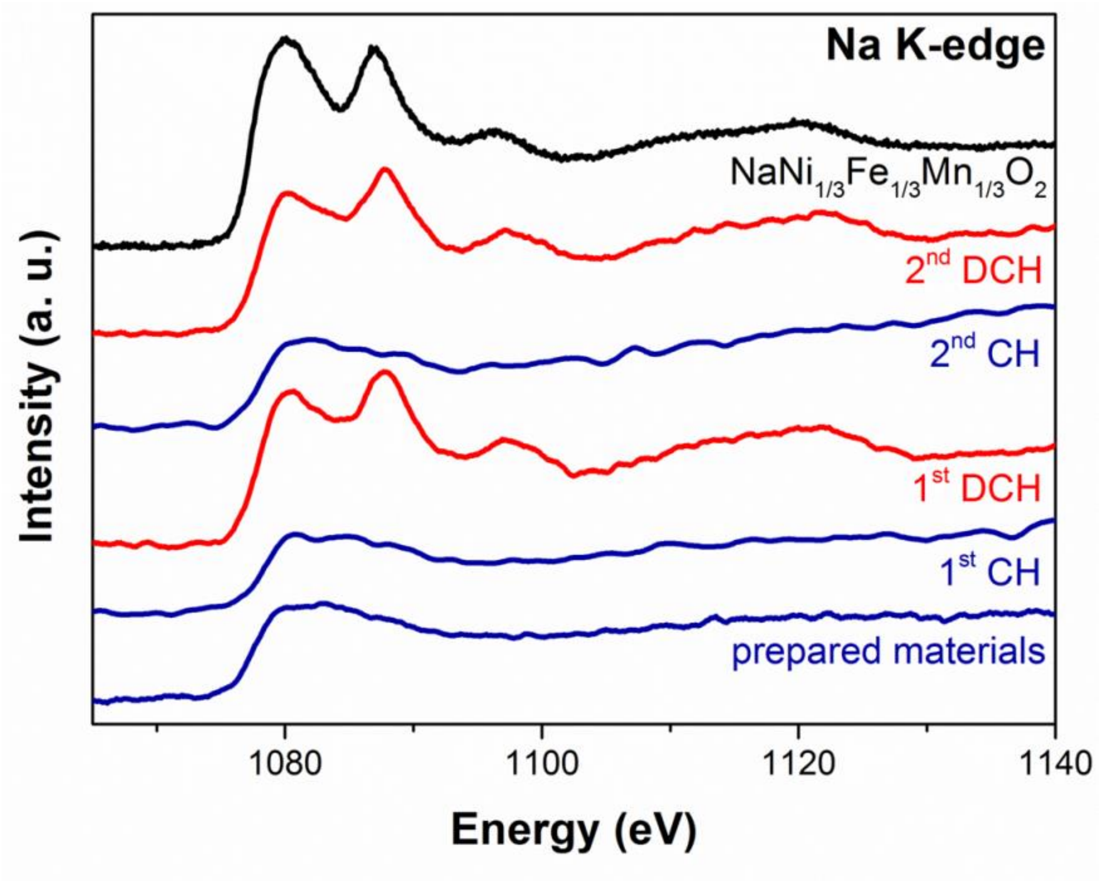


**Supplementary Figure 10** Selected Na K-edge XANES spectra of partially dehydrated Na-bir at the 1st charge, 1st discharge, 2nd charge, and 2nd discharge with reference $NaNi_{1/3}Fe_{1/3}Mn_{1/3}O_2$ spectra.

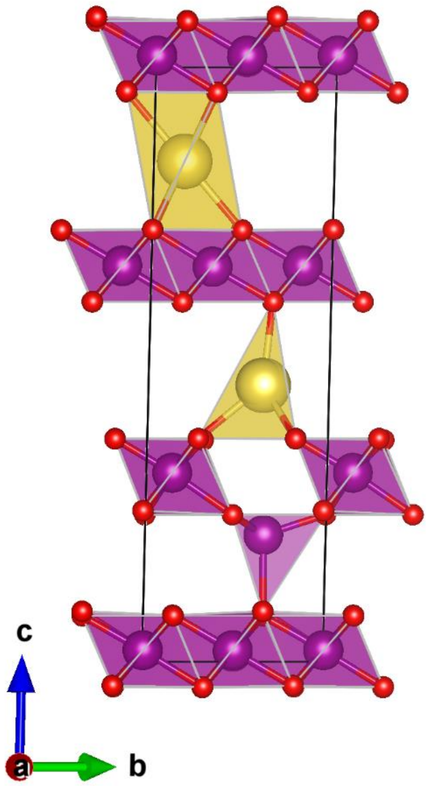


**Supplementary Figure 11** Relaxed atomic structure of Mn-migrated Na-bir using first-principles calculation.

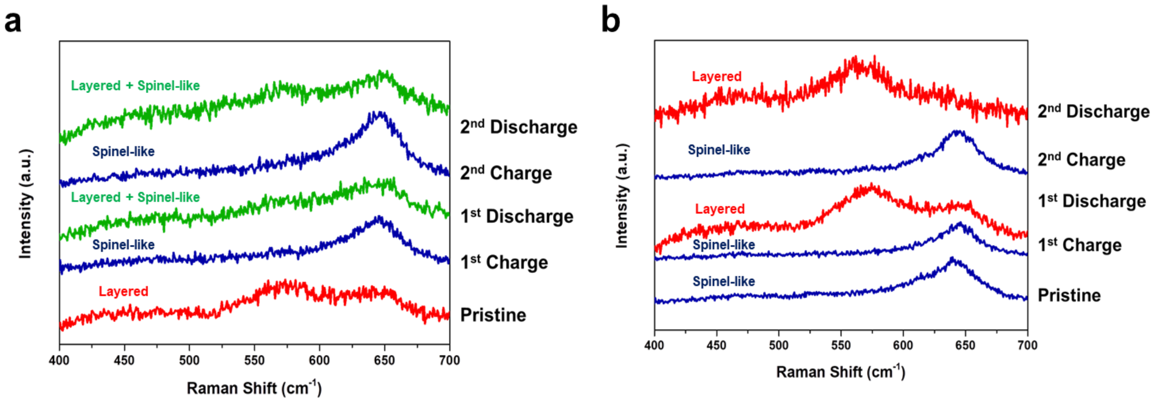


**Supplementary Figure 12 Variations of Raman spectra of hydrated and partially dehydrated Na-birs.** *Ex situ* Raman spectra of (a) hydrated Na-bir and (b) partially dehydrated Na-bir at pristine, 1$^{st}$ charged, 1$^{st}$ discharged, 2$^{nd}$ charged and 2$^{nd}$ discharged state.

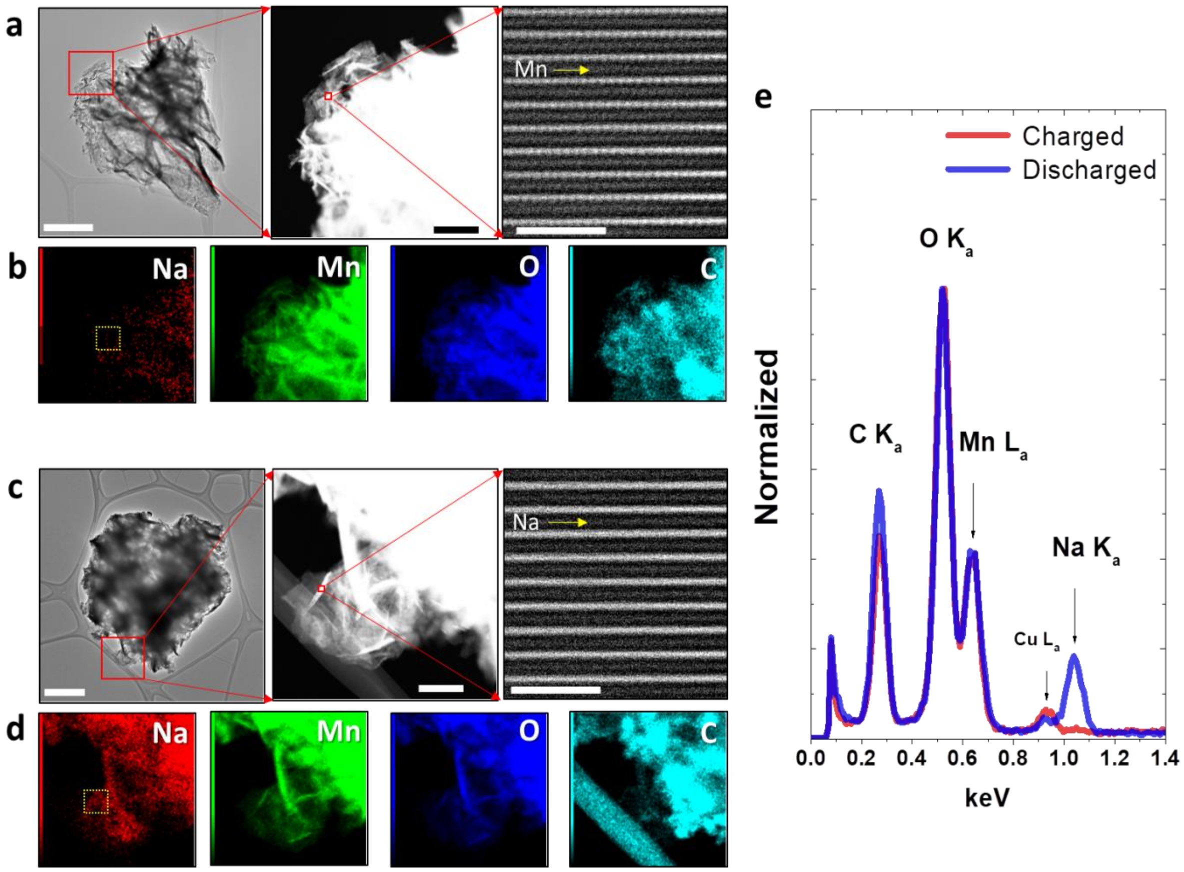


**Supplementary Figure 13 Extra structural and chemical analysis of partially dehydrated Na-bir samples.** Bright-field (BF) TEM images of (a) charged and (c) discharged samples, respectively. The regions of interest denoted by red boxes in the BF-TEM images were consecutively observed by ADF-STEM imaging at low-magnification and high-resolution. It is clearly observed again that Mn ions (heavier than Na ion thus showing brighter contrast) are migrated into Na layer due to desodiation, while Na ions (lighter than Mn ion thus showing weak contrast) are refilled into the interlayer after discharging. EDX mapping for the regions corresponding to the low-magnification ADF-STEM images was simultaneously carried out as shown in (b) and (d) for Na (red), Mn (green), O (blue), and C (cyan), respectively. (e) From the comparison of EDX spectra for the two respective regions marked by yellow dotted boxes in (b) and (d), it is corroborated that Na ions were almost removed and refilled in the interlayer during charge and discharge processing (see the Na $K_a$ peak in (e)), while the Mn and O were not noticeably altered. Note that strong carbon X-ray signals were attributed to the unremoved binder (undesirably begetting serious hindrance to high-resolution STEM imaging) and small copper ones to Cu TEM grid for sampling.

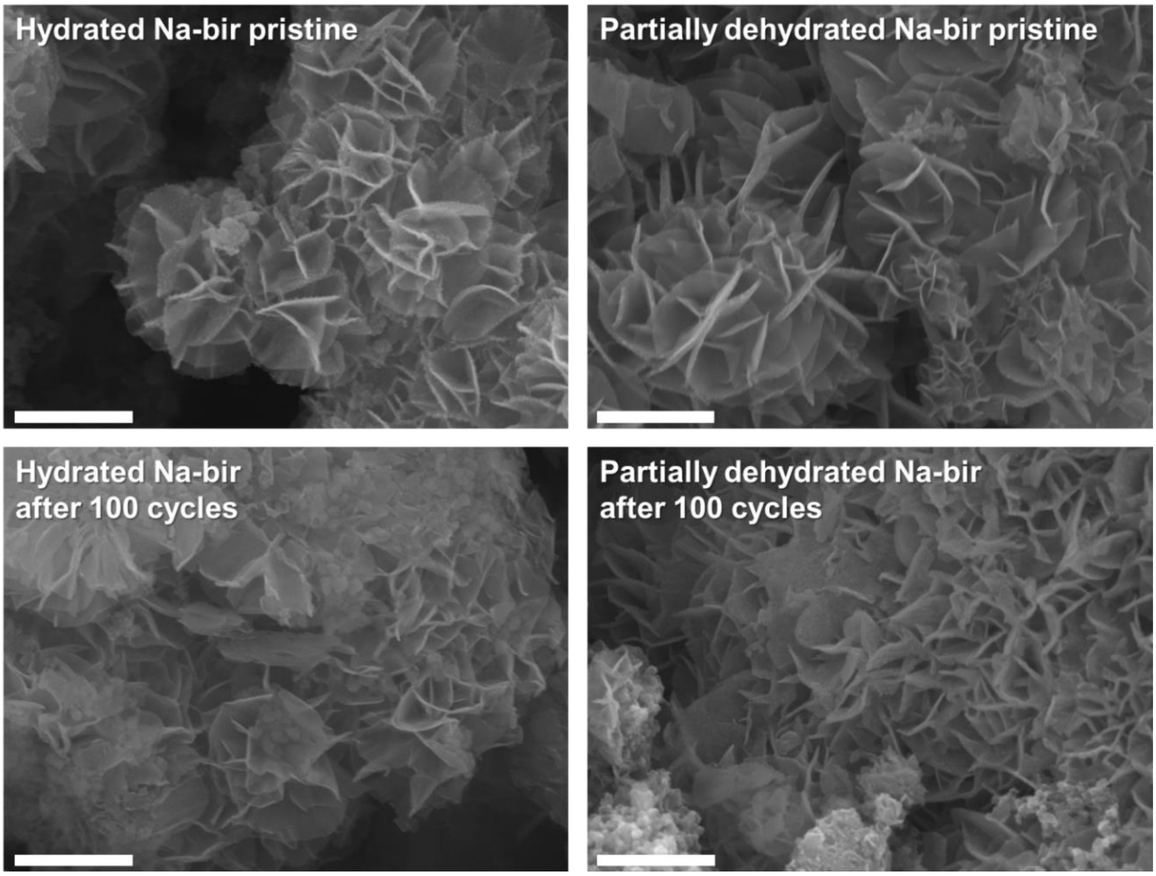


**Supplementary Figure 14** SEM images of hydrated and partially dehydrated Na-bir electrode at pristine state and after 100 cycles. (scale bar = 1μm)

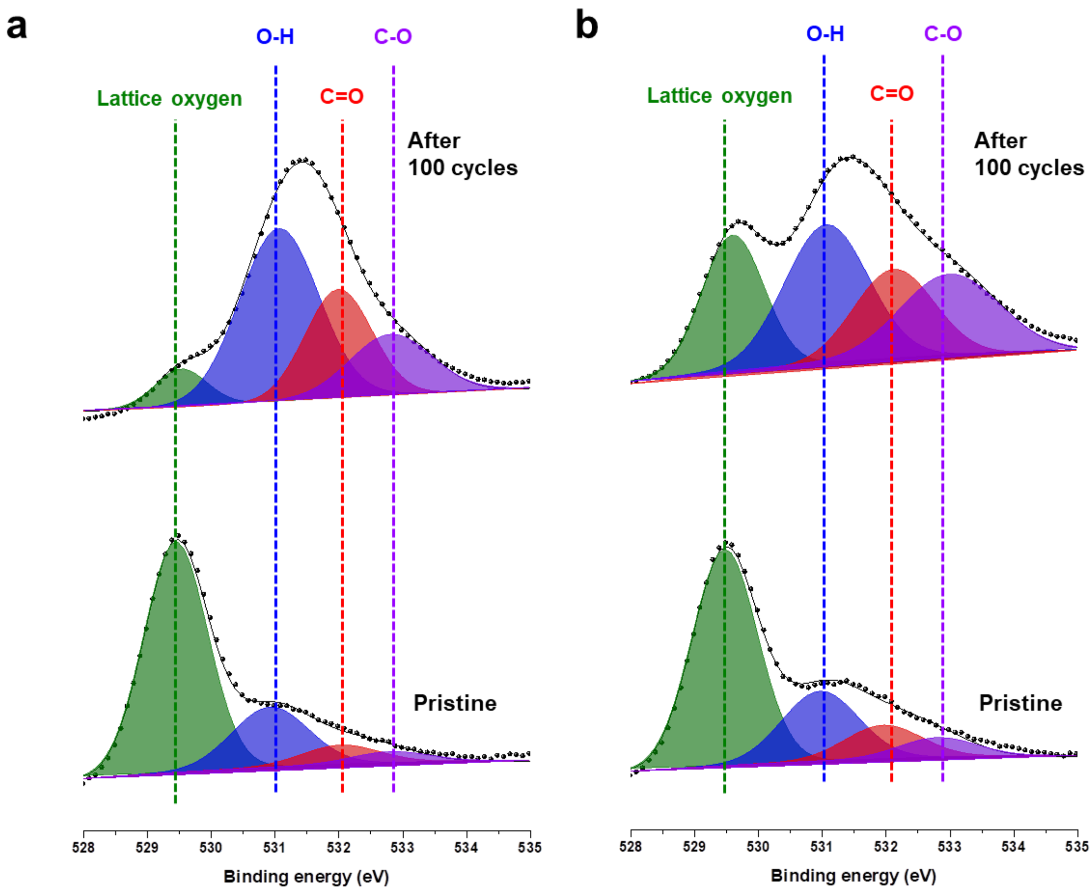


**Supplementary Figure 15 XPS analysis of pristine and after 100 cycles of hydrated and partially dehydrated Na-bir electrodes.** XPS O1s spectra of (a) hydrated Na-bir and (b) partially dehydrated Na-bir electrode at pristine state and after 100 cycles.

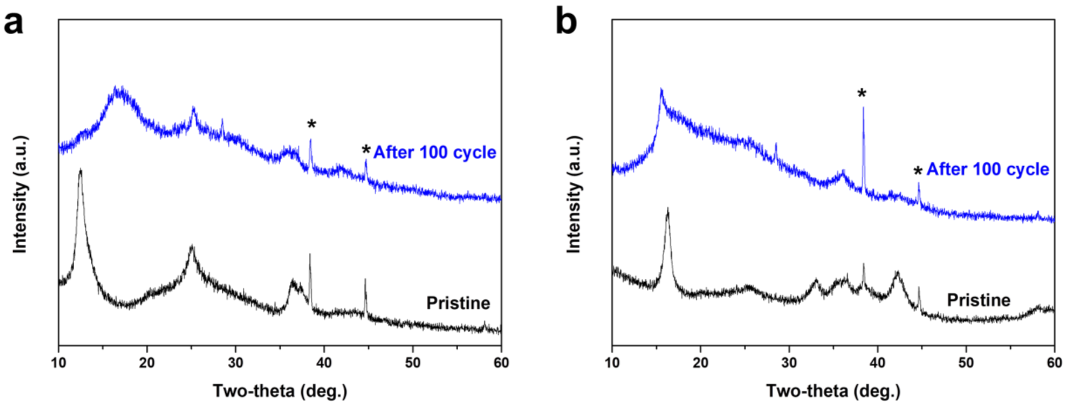


**Supplementary Figure 16 XRD analysis of pristine and after 100 cycles of hydrated and partially dehydrated Na-bir electrodes.** *Ex situ* XRD patterns of (a) hydrated Na-bir and (b) partially dehydrated Na-bir after 100 cycles

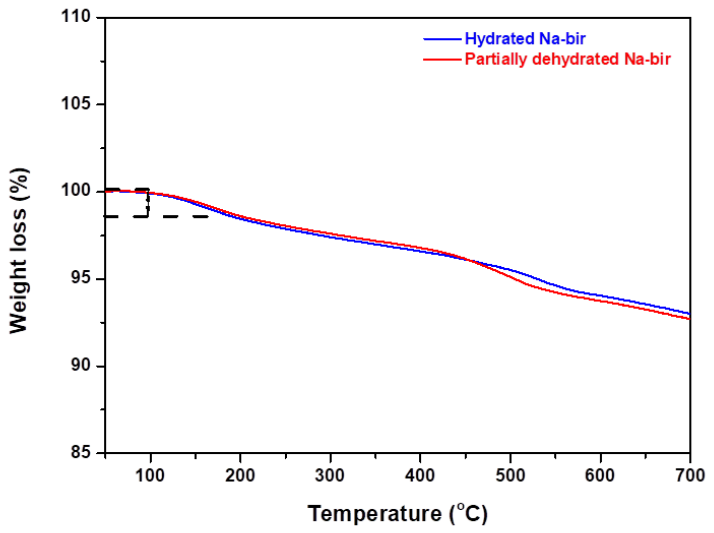


**Supplementary Figure 17** TGA curves of hydrated and partially dehydrated Na-bir electrode after 100 cycles

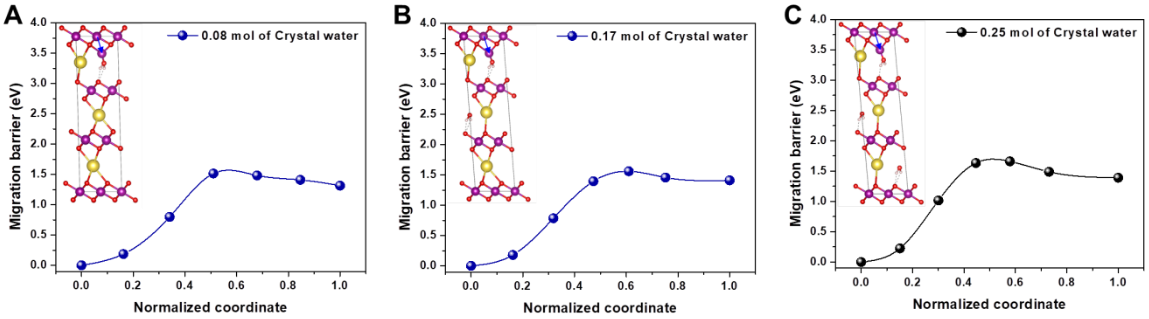


**Supplementary Figure 18** Migration barriers of Mn ions from an octahedral site in Mn layer to tetrahedral site in Na layer for (a) $Na_{0.27}MnO_2{\cdot}0.08H_2O$, (b) $Na_{0.27}MnO_2{\cdot}0.17H_2O$, and (c) $Na_{0.27}MnO_2{\cdot}0.25H_2O$.

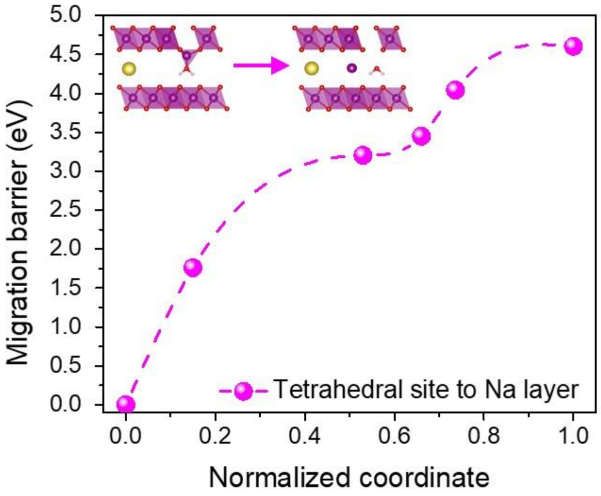


**Supplementary Figure 19** Migration barrier of Mn ion from tetrahedron with crystal water to the Na layer in $Na_{0.27}MnO_2·0.08H_2O$.

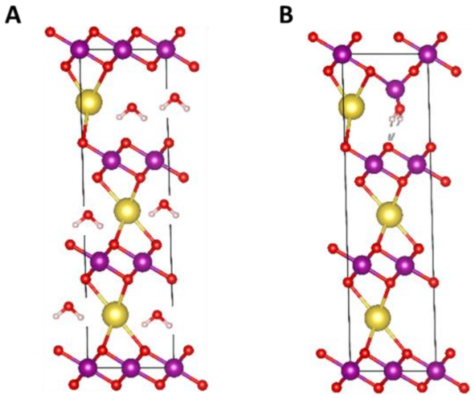


**Supplementary Figure 20 Calculations on extraction energy of crystal water from hydrated and partially dehydrated Na-birs.** Crystal structure models for calculating the extraction energy of crystal water from (a) hydrated Na-bir and (b) partially dehydrated Na-bir.

**Supplementary Table 1** Concentration of elements in hydrated Na-bir obtained from ICP-AES result (ppm=mg $kg^{-1}$)

| Na | Mn |
|---|---|
| 620669.35 | 71115.00 |

**Supplementary Table 2** Concentration of Mn deposited on Na anodes after 100 cycles (unit: ppm=mg $L^{-1}$)

| | **Mn** |
|---|---|
| Pure Na metal | - |
| Na metal cycled with Hydrated Na-bir | 1.77 |
| Na metal cycled with Partially dehydrated Na-bir | 0.41 |

**Supplementary Table 3** Extraction energy of crystal water between Mn layer and crystal water bonded to migrated Mn

| | $H_2O$ extraction energy |
|---|---|
| Crystal water between Mn layers | 0.1973 eV |
| Crystal water bonded to migrated Mn | 0.8978 eV |

**Supplementary Note 1 Relationship between reversible structural changes and kinetic properties**

The relationship between the corresponding reversible phase transformation and kinetic properties of Na-bir were demonstrated by galvanostatic intermittent titration technique (GITT) analysis (Supplementary Figure 4). As shown, it is observed that partially dehydrated Na-bir shows higher $Na^+$ ion coefficient than that of hydrated Na-bir. Interestingly, the diffusion coefficient values of both hydrated and partially dehydrated Na-bir were maximized as voltage increased where the layered structure changed into spinel-like structure. This is because the ion transport in spinel or spinel-like structure is usually faster than layered structure due to three dimensional interstitial diffusion pathways.

**Supplementary Note 2 Comparison of electrochemical performances of hydrated, partially dehydrated, and fully hydrated Na-birs.**

As shown in Supplementary Figure 5 and 6, 1st discharge capacity of fully dehydrated Na-bir at 0.1C is 176 mAh $g^{-1}$ which is similar to that of hydrated Na-bir, but lower than that of partially dehydrated Na-bir. Furthermore, its capacity after 100 cycles just comes to ~30 mAh $g^{-1}$ which is 17% of its 1st discharge capacity. Furthermore, its rate capability is much inferior when it is compared to that of hydrated Na-bir and partially dehydrated Na-bir. It only shows 36.26 mAh $g^{-1}$ at 5C which corresponds to 20% of the discharge capacity at 0.1C, while hydrated Na-bir and partially dehydrated Na-bir maintain 38% and 44%. These results demonstrate that fully dehydrated Na-bir exhibits inferior electrochemical performances to hydrated Na-bir. By conducting these additional experiments, we confirmed that the effect of crystal water contents in birnessite structure is very important to regulate its electrochemical performances.

**Supplementary Note 3 Calculations of projected density of states and Bader charge.**

We calculated the projected density of states and Bader charge for the both cases as shown in Supplementary Figure 9. The Mn ion in the original octahedral site shows typical $Mn^{4+}$ that spin-up states are partially occupied and the other spin- and spin-down states are unoccupied with larger gap between $t_{2g}$ and $e_g$ levels. While, the PDOS of the migrated Mn ion shows narrowed gap between $t_2$ and $e$ levels due to the electronic configuration of tetrahedral coordination, and the occupied area of the migrated Mn ion increased comparing to that of the original Mn ion. It is also revealed from the Bader charge analysis that the electronic charge of the migrated Mn ion in the tetrahedral site is larger than that of the original Mn ion in the octahedral site. This means that the migrated tetrahedral Mn ion has more electrons toward $3^+$ valence state comparing to the original octahedral Mn ion in $4^+$ valence state.

**Supplementary Note 4 Variations of Raman spectra of hydrated and partially dehydrated Na-birs.**

In Raman spectra, similarity of spectral signal suggests that the materials have a similar structure. Basically, Raman spectra of layered birnessite shows bands at ~575 $cm^{-1}$ and ~650 $cm^{-1}$ arising from the Mn-O bond stretching vibration from the basal plane of [$MnO_6$] laminates while spinel phase shows a sharp band at ~650 $cm^{-1}$ which can be assigned to A1g mode of tetrahedrally coordinated Mn-O. The Raman spectra of hydrated Na-bir at pristine state exhibit bands at ~575 $cm^{-1}$ and ~650 $cm^{-1}$ which demonstrates that the pristine state has layered structure. After it charged, band at ~575 $cm^{-1}$ disappeared and only sharp band at ~650 $cm^{-1}$ remained. Thus, it is observed that hydrated Na-bir also go through phase transformation from layered to spinel-like phase when it is charged up to 4.3V as similar to partially dehydrated Na-bir. However, although the band at ~575 $cm^{-1}$ appears again after discharged, the intensity of the band at ~650 $cm^{-1}$ is still stronger than that of band at ~575 $cm^{-1}$ in hydrated Na-bir. This means that the layered structure evolved again during discharge, but there is still spinel-like structure in hydrated Na-bir. This change is also maintained in the $2^{nd}$ cycle, indicating that the hydrated Na-bir exhibits lower structural reversibility during electrochemical reaction.

**Supplementary Note 5 Morphology changes of hydrated and partially dehydrated Na-birs after 100 cycles.**

Morphology of hydrated Na-bir and partially dehydrated Na-bir in electrode shows microflower-like morphology as shown in HR-TEM images in manuscript. Herein, small round-shape particles are conductive carbonaceous additive added during electrode preparation. After 100 cycles, both hydrated Na-bir and partially dehydrated Na-bir well maintains its microflower-like morphology as shown in Supplementary Figure 14. Therefore, collapse of morphology is not significant for degradation of electrochemical performances.

**Supplementary Note 6 XPS analysis of pristine and after 100 cycles of hydrated and partially dehydrated Na-bir electrodes.**

To study the CEI layer of hydrated Na-bir and partially dehydrated Na-bir, O1s spectra at pristine state and after 100 cycles of both samples were analyzed. As for pristine electrode, both O1s spectra shows the most intense lattice oxygen peak with several oxygen moieties like O-H, C=O from $Na_2CO_3$ and C-O which may be generated due to the reaction with atmospheric moisture and $CO_2$. After 100 cycles, lattice oxygen peak intensity is decreased while the peaks corresponding to O-H, C=O and C-O become intense. The change in the O1s spectra is indicative of CEI layer formation during cycling. However, it is observed that relative peak intensity of lattice oxygen from partially dehydrated Na-bir is much higher than that of hydrated Na-bir. Considering that the limited depth of XPS analysis, the difference in lattice oxygen peak intensity is associated with different CEI thickness. Therefore, it is clear that hydrated Na-bir is much more sensitive toward electrolyte decomposition compared to partially dehydrated Na-bir.

**Supplementary Note 7 Mn ion dissolution in hydrated and partially dehydrated Na-birs after 100 cycles.**

Dissolution and migration of Mn species from Mn-containing cathodes to anode have been considered to be an important issue. This phenomenon is irreversible and is related to a gradual capacity fading of Mn-containing cathode material. Generally, it is accepted that $Mn^{2+}$ species are dissolved into electrolyte as a result of disproportionation reaction of $Mn^{3+}$ in Mn-containing cathode material as follows.

$$2Mn^{3+} \rightarrow Mn^{2+} + Mn^{4+}$$

As a result of disproportionation reaction, $Mn^{4+}$ which is insoluble in electrolyte remains in the material while $Mn^{2+}$ dissolves into electrolyte. Dissolved $Mn^{2+}$ species finally diffuse and are deposited on anode side during electrochemical reaction resultantly leading to a gradual capacity fading. Hence, the concentration of deposited Mn species on Na anode is measured using ICP-AES and the corresponding results are presented. Significantly higher amount of Mn (~1.77 ppm) is observed on the surface of Na metal cycled with hydrated Na-bir, while the Na metal cycled with partially dehydrated Na-bir only has 0.41 ppm of Mn after 100 cycles.

**Supplementary Note 8 XRD analysis of hydrated and partially dehydrated Na-bir electrodes at pristine and after 100 cycles.**

To investigate the structural stability of both samples, *ex situ* XRD analyses were conducted at pristine state and after 100 cycles. As observed in Supplementary Figure 16, pristine electrodes of hydrated Na-bir and partially dehydrated Na-bir show intense (002) peaks at $12.5^{o}$ and $16.3^{o}$, respectively. The difference in peak position between pristine hydrated and partially dehydrated Na-bir electrodes well corresponds to that observed in the powder XRD patterns, which stems from the different crystal water contents. For hydrated Na-bir after 100 cycles, the (002) peak almost diminished and a broad peak around $17.06^{o}$ appeared. However, the characteristic (002) peak is well preserved in partially dehydrated Na-bir electrodes even after 100 cycles. This observation demonstrates that hydrated Na-bir undergoes a drastic structure collapse. Thus, controlling crystal water contents in birnessite structure can be regarded as a key to facilitate the reversible phase transformation and stabilize the structure.

**Supplementary Note 9 TGA analysis of hydrated and partially dehydrated Na-bir electrodes after 100 cycles.**

To verify the remaining crystal water contents in hydrated and partially dehydrated Na-bir after electrochemical cycling, TGA analyses were conducted for the electrode after 100 cycles. As shown in Supplementary Figure 17, the weight loss up to 100$^oC$ is not significant from both electrodes, which demonstrates that loss of adsorbed moisture from the electrode is negligible. From 100$^oC$ to 170$^oC$, the weight loss of both samples is nearly 1 wt% which comes from crystal water loss. This result demonstrates that remaining crystal water contents in both samples is similar after 100 cycles.

**Supplementary Note 10 Calculations on extraction energy of crystal water from hydrated and partially dehydrated Na-birs.**

In order to investigate the stability of crystal water in birnessite structure, we calculated the extraction energy of crystal water considering the environment (Supplementary Figure 20). The stabilized model structure for first-principles calculations tells us that one crystal water prefers to interact with another rather than the Mn ions migrated to tetrahedral sites and any other constituting element of framework if the amount of crystal water is above that in partially dehydrated Na-bir. So, we could assume the crystal water between Mn layers for hydrated Na-bir, while the crystal water bonded to migrated Mn was used for the calculation on partially dehydrated Na-bir.

**Supplementary Note 11 Extraction energy of crystal water between Mn layer and crystal water bonded to migrated Mn.**

As shown in Supplementary Table 3, the extraction energy of crystal water between Mn layers is 0.1973 eV, whereas the crystal water bonded to Mn ions migrated to tetrahedral sites has 0.8938 eV. From these results, it is revealed that the crystal water in partially dehydrated Na-bir is much more stable than that in hydrated Na-bir. This observation partially supports the reason why partially dehydrated Na-bir can present higher stability and capacity retention after 100 cycles.
